# SPP1 mRNA Expression Is Associated with M2 Macrophage Infiltration and Poor Prognosis in Triple-Negative Breast Cancer

**DOI:** 10.3390/cimb46120806

**Published:** 2024-11-25

**Authors:** Yu-Chia Chen, Chia-Ching Chen, Rong-Fu Chen, Hsin-Hung Chen, Po-Ming Chen

**Affiliations:** 1 Division of General Surgery, Department of Surgery, Kaohsiung Veterans General Hospital, Kaohsiung 813414, Taiwan; ycchen@vghks.gov.tw; 2 Department of Emergency Medicine, Chang Bing Show Chwan Memorial Hospital, Changhua 505, Taiwan; c11g@hotmail.com; 3 Division of Plastic Surgery, Department of Surgery, Kaohsiung Medical University Hospital, Kaohsiung 807, Taiwan; dr.chenrf@gmail.com; 4 Regenerative Medicine and Cell Therapy Research Center, Kaohsiung Medical University, Kaohsiung 807, Taiwan; 5 Department of Medical Education and Research, Kaohsiung Veterans General Hospital, No. 386, Dazhong 1st Road, Zuoying District, Kaohsiung 813414, Taiwan; 6 Research Assistant Center, Show Chwan Memorial Hospital, No. 542, Section 1, Zhongshan Road, Changhua 500, Taiwan; 7 Department of Nursing, Central Taiwan University of Science and Technology, Taichung 406, Taiwan

**Keywords:** triple-negative breast cancer, SPP1, prognosis

## Abstract

(1): Triple-negative breast cancer (TNBC) is an especially aggressive form of breast cancer defined by a poor prognosis and a lack of effective treatment options. There is a pressing need for validated predictive and prognostic biomarkers to assist in making treatment decisions and improve the prognostic accuracy for patients with this challenging disease. (2): We analyzed the RNA-seq data from three TNBC tissue samples alongside their corresponding normal tissues. Gene set enrichment analysis (GSEA) identified potential pathways. Additionally, we examined SPP1 mRNA expression datasets available in the Kaplan–Meier plotter and investigated the SPP1 protein expression patterns in our own tissue microarray cohort via immunohistochemistry. (3): The results revealed that genes associated with the Toll-like receptor signaling pathway showed a significant increase in activity in TNBC tissues when compared to normal breast tissues. Furthermore, *SPP1* expression was found to be elevated in the TCGA TNBC dataset and correlated with a poor prognosis. This pattern was corroborated at the protein level in our TNBC tissue cohort; however, SPP1 protein expression did not demonstrate a significant impact on survival. Notably, *SPP1* mRNA expression was strongly linked to tumor-associated macrophages (TAMs), particularly the M2 macrophage subtype, indicating a substantial association in the context of TNBC. (4): Our research highlights the significance of *SPP1* mRNA as a key prognostic indicator and a potential molecular responder for TNBC treatment utilizing targeted therapies that focus on Toll-like receptor signaling pathways.

## 1. Introduction

Breast cancer is the most common type of cancer in women, accounting for 30% of all cancer cases in females. Although it can affect women of all ages, it is particularly prevalent among younger women. Over recent decades, there has been a gradual yet steady increase in the incidence of breast cancer, with rates rising by approximately 0.3% per year. Despite this upward trend in diagnoses, significant strides have been made in reducing mortality rates in the United States. As of 2017, the death rate from female breast cancer has seen a remarkable decline of 40% since its peak in 1989 in the United States [1]. In 2020, Asia reported the lowest breast cancer age-standardized incidence rate among global regions, standing at 36.8 per 100,000 females. This rate was notably lower compared to other regions, including Africa (40.7 per 100,000), Latin America and the Caribbean (51.9 per 100,000), Europe (74.3 per 100,000), Oceania (87.8 per 100,000), and North America (89.4 per 100,000) [2]. Asia recorded the lowest age-standardized mortality rate for breast cancer globally, standing at 11.6 per 100,000 females, including Oceania (13.2 per 100,000), Latin America and the Caribbean (13.5 per 100,000), Europe (14.8 per 100,000), North America (16.9 per 100,000), and Africa, which had the highest rate at 19.4 per 100,000 females [2].

Hormone therapy, which targets the progesterone receptor (PR) and estrogen receptor (ER), along with targeted therapy, is commonly recommended for breast cancer tumors that express hormone receptors (HRs) or human epidermal growth factor receptor 2 (HER2). These therapies work by interfering with the hormonal signals or growth factors that fuel the growth and spread of certain types of breast cancer [3]. Estrogen receptors (ERs) and progesterone receptors (PRs) are key biological markers that play a critical role in breast cancer diagnosis and treatment decisions. Approximately 70–80% of breast carcinomas express estrogen receptors, making ER-positive breast cancer one of the most common subtypes of the disease [4,5]. The human epidermal growth factor receptor 2 (HER2) oncoprotein is a significant biomarker in breast cancer, with overexpression observed in approximately 20–25% of primary breast carcinomas. This overexpression is typically identified through immunohistochemistry (IHC) staining, which is performed using standardized and approved reagents, testing protocols, and a specific scoring algorithm to ensure the accuracy of the assessment. HER2-positive breast cancers tend to be more aggressive, characterized by faster growth and a higher likelihood of recurrence, which makes identifying the HER2 status critical for determining the most effective treatment options [5]. In cases where the HER2 status is not clearly positive or negative—referred to as HER2-equivocal—further testing is required to clarify the status. Approximately 10–20% of breast carcinomas that are initially classified as HER2-equivocal through IHC are later found to be HER2-amplified using fluorescence in situ hybridization (FISH) testing, ensuring that patients who would benefit from HER2-targeted therapies, such as trastuzumab or pertuzumab, receive the appropriate treatment [6].

The Breast Cancer Conference introduced a new classification system for breast cancer based on molecular subtypes, providing a more refined approach to diagnosis and treatment. These subtypes include the following. (1) Luminal A: Characterized by the presence of estrogen receptors (ER+) and/or progesterone receptors (PR+), this subtype is hormone receptor-positive and typically has the best prognosis. (2) Luminal B: This subtype also expresses ER+ and/or PR+, but it can also include HER2 positivity (HER2+), representing a more aggressive form of hormone receptor-positive breast cancer. (3) HER2 overexpression: This subtype lacks ER and PR expression (ER−, PR−) but shows overexpression of HER2, leading to a more aggressive cancer type. (4) Basal-like or triple-negative breast cancer (TNBC): This subtype is negative for ER, PR, and HER2 (ER−, PR−, HER2−) and is often the most difficult to treat, as it does not respond to hormone therapies or HER2-targeted treatments. Among these subtypes, Luminal A is the most commonly diagnosed, accounting for 59.0% of cases, and has the highest survival rates due to its responsiveness to hormone therapies. In contrast, triple-negative breast cancer (TNBC) is associated with the poorest survival outcomes, as it tends to be more aggressive and lacks targeted treatment options, requiring alternative approaches like chemotherapy [7].

Phosphoprotein 1 (SPP1), commonly referred to as osteopontin, is a highly versatile and multifunctional secreted glycoprotein that undergoes phosphorylation. Osteopontin plays a pivotal role in various biological processes due to its ability to interact with different cell types and molecular pathways [8,9,10]. In addition to being found in tissues, SPP1 is present in various body fluids, such as serum, bovine milk, and human urine, indicating its widespread physiological roles. At the cellular level, however, SPP1 expression is more restricted, being primarily produced by specific cell types. These include osteoblasts, which are involved in bone formation; fibroblasts, which play a role in connective tissue maintenance; and immune-related cells such as macrophages, dendritic cells, lymphoid cells, and mononuclear cells [8,9,10]. The expression of SPP1 in tumor-associated macrophages (TAMs) has been linked to unfavorable clinical outcomes in lung adenocarcinoma. Specifically, high levels of SPP1 expression in these TAMs are associated with a poor prognosis, as they play a role in promoting tumor progression and creating an immunosuppressive tumor microenvironment [8]. SPP1 (osteopontin, OPN) has also been found to be overexpressed in ovarian tumor tissues, with higher levels of SPP1 expression linked to poorer survival outcomes in patients. Notably, elevated SPP1 expression is positively associated with increased infiltration of various immune cells, including CD4+ T cells, CD8+ T cells, macrophages, neutrophils, and dendritic cells, suggesting that SPP1 plays a significant role in shaping the immune environment of ovarian cancer [11]. The SPP1 protein has the potential to act as a prognostic biomarker for patients with bladder cancer [12].

This study investigated the role of SPP1 in the prognosis of triple-negative breast cancer (TNBC) and its potential involvement in immune regulation. While SPP1 has been linked to cancer cell growth and immune processes, its specific impact on the TNBC prognosis remains unclear, and the interactions between SPP1 and immune regulation in TNBC have yet to be fully understood. To address this, we analyzed RNA-Seq data from three TNBC samples using gene set enrichment analysis (GSEA). The analysis revealed that the Toll-like receptor signaling pathway, including genes like *SPP1*, was among the most activated pathways. Additionally, we studied the *SPP1* gene expression profile in BRCA and adjacent normal breast tissue from the Cancer Genome Atlas (TCGA). Clinically relevant SPP1 protein expression was also examined through immunohistochemistry in tissue arrays from our BRCA cohort (*n* = 282). Our findings revealed a novel association between *SPP1* mRNA expression and TNBC recurrence and prognosis. Finally, using TIMER2.0, we explored the correlation between *SPP1* mRNA expression and macrophage infiltration in TNBC.

## 2. Materials and Methods

### 2.1. Patients

Clinical samples were obtained from patients diagnosed with breast cancer, all of whom provided informed consent prior to participation in the study. These patients agreed to undergo surgical procedures at Kaohsiung Veterans General Hospital, a prominent medical facility located in Kaohsiung, Taiwan. This research was conducted in accordance with ethical standards and was granted approval by the Institutional Review Board (IRB) of Kaohsiung Veterans General Hospital, ensuring that all ethical considerations were addressed. The specific IRB approval number for this study is KSVGH22-CT1-08. In this study, two different sets of breast cancer tissue microarrays were used among a total of 282 patients with breast cancer diagnosed between 1991 and 2011.

### 2.2. RNA-Seq

RNAs extracted from three TNBC tissue samples, alongside their corresponding normal tissues, were sent to Genomics (Taipei, Taiwan) for analysis, library construction, purification, library QC and quantitation before use for transcriptome RNA sequencing experiments. The fragments per kilobase of transcript per million mapped reads (FPKM) calculation aims to determine the transcript length and overall sequencing quantity.

### 2.3. Immunohistochemistry and Scoring

For each patient, representative tissue cores from both the BRCA and adjacent normal sections were carefully collected and processed into tissue microarray slides. The slides were heated in a 60 °C oven for 60 min and then transferred to a xylene bath for three changes for 5 min each. Excess liquid was shaken off, and the slides were sequentially placed in fresh 100% ethanol, 95% ethanol, 75% ethanol, and 50% ethanol for 5 min each. Afterward, the slides were rinsed under gently running tap water for 5 min and then placed in a PBS wash bath for 30 min. Immunohistochemistry (IHC) staining was performed using a primary mouse anti-human SPP1 monoclonal antibody (osteopontin/OPN/SPP1 antibody, AKm2A1: sc-21742; Santa Cruz Biotechnology, Inc. , Dallas, TX, USA) applied at a concentration of 1:200. Amplifier for Mouse and Rabbit (Cell Marque, 954D-21-RUO, Rocklin, CA, USA), HRP polymer (954D-22-RUO, Cell Marque, Rocklin, CA, USA), DAB chromogen (Epredia, TA-125-QHDX, Kalamazoo, MI, USA), and hematoxylin (Leica, 3801552, Barrington, IL, USA) were used for counterstaining. The sections were mounted using Surgipath Micromount (Leica, 3801731, Barrington, IL, USA) mounting medium. To objectively evaluate the immunostaining results, we performed whole-slide scanning with computer-assisted analysis [13]. The slides were scanned at 200× magnification using a Panoramic DESK II DW slide scanner (3DHISTECH, Budapest, Hungary). For analysis, we used CellQuant and PatternQuant software (version 2.4.0.119028, 3DHISTECH, Budapest, Hungary). Briefly, PatternQuant was trained to identify regions of interest, which were subsequently analyzed by CellQuant to calculate the H-score. The H-score was determined by multiplying the immuno-intensity by the staining percentage, yielding a range from 0 to 300. The immuno-intensity was categorized as follows: 0 (no staining), 1 (faint staining), 2 (moderate staining), and 3 (intense staining). The staining percentage values ranged from 0% to 100%. Both parameters were automatically calculated by CellQuant, which only analyzed regions of interest identified by PatternQuant. To ensure the accuracy, the original scanned slides and analyzed images were manually reviewed using semi-quantitative evaluation.

### 2.4. Correlation Analysis

We employed the “Gene_Corr” module of TIMER2.0 (Tumor Immune Estimation Resource, version 2) (http://timer.cistrome.org/, accessed on 1 October 2024) to explore the relationship between the target genes and the BRCA prognosis [14]. Additionally, through the “Gene_DE” module of TIMER2.0, we examined the expression of *SPP1* and compared its levels between the tumor tissues and their corresponding normal tissues across different cancers in the TCGA dataset.

### 2.5. Statistical Analysis

The Kaplan–Meier plotter (https://kmplot.com/, accessed on 1 October 2024) was used for the survival analyses, as previously described [15,16]. The expression levels of the *SPP1* gene were extracted from the gene expression profile data, and the samples were stratified into high- and low-expression groups based on the median value. Prognostic analyses were conducted using log-rank tests, with *p*-values less than 0.05 considered statistically significant. The immunohistochemistry (IHC) scores of SPP1 were analyzed to determine the expression differences between the BRCA tissues and their corresponding normal tissues, employing a paired sample *t*-test. The Kaplan–Meier plotter was utilized to examine the relationship between SPP1 expression and clinical factors such as the cancer stage, the ER, PR, and HER2 status, along with the survival time. A *p*-value of less than 0.05 was considered to indicate statistical significance. All the statistical analyses were performed using SPSS 18.0 software (SPSS, Inc., Chicago, IL, USA).

## 3. Results

### 3.1. Toll-like Receptor Signaling Pathway Is Enriched in the Three TNBC RNA-Seq Data

**Figure 1:**
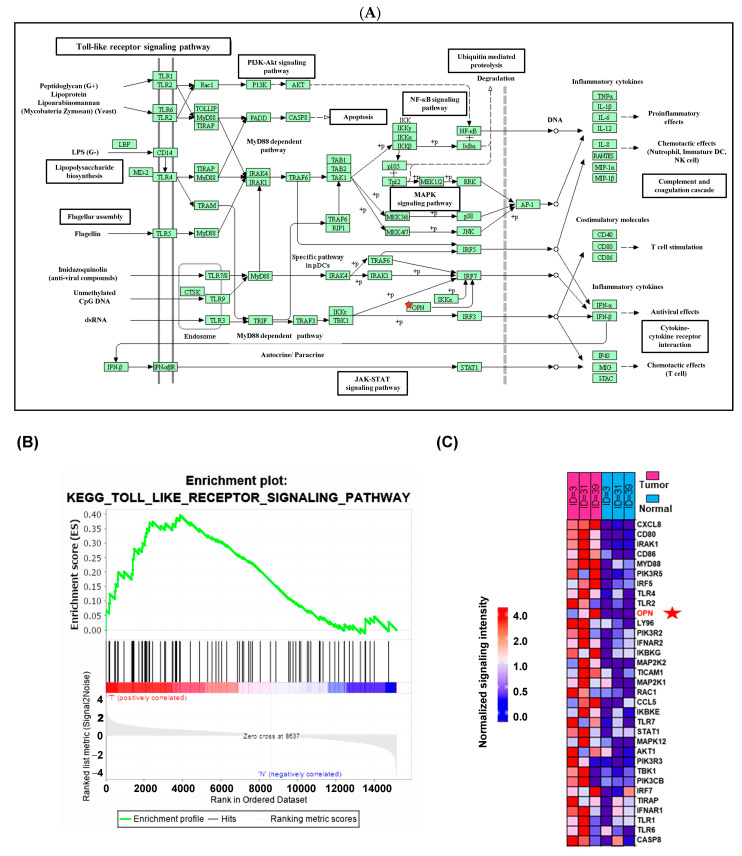
GSEA results showing the Toll-like receptor signaling pathway is a differentially enriched pathway in the three TNBC. (**A**) KEGG pathway annotations of the Toll-like receptor signaling pathway. (**B**) NES (normalized enrichment score) of each Toll-like receptor signaling pathway-related genes. (**C**) Data for the positive association genes are visualized in a heat map. SPP1 (OPN) is marked with an asterisk.

To investigate the problem of prognostic markers in TNBC, we randomly selected three tumor–normal paired tissue samples from three TNBC patients in our cohort to support a robust statistical analysis, which are known for their stage and poor differentiation tendencies (Table 1). To identify differentially expressed (DE) genes and signaling pathways associated with cancer progression in TNBC, we conducted RNA sequencing and utilized gene set enrichment analysis (GSEA) version 4.3.3 software for Windows. This analysis compared the gene expression and pathway profiles between the TNBC tissues and their corresponding normal tissues. The Toll-like receptor signaling pathway was enriched in the three TNBC pairs (Figure 1A,B). Heatmaps of the Toll-like receptor signaling pathway genes were made in three TNBC tissues and their corresponding normal tissues (Figure 1C).

### 3.2. SPP1 mRNA Expression in Common Cancers

**Figure 2:**
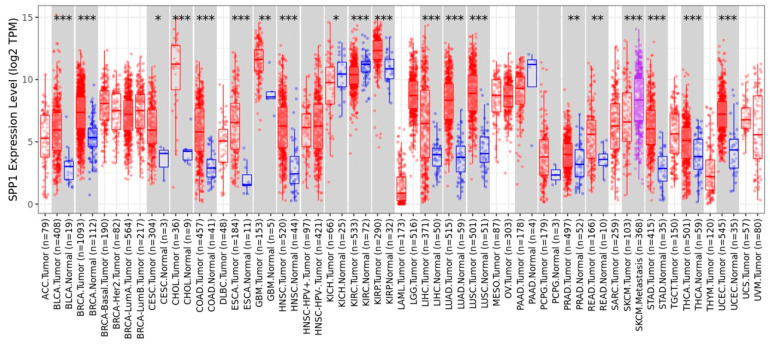
The mRNA expression of *SPP1* across various cancer types and their corresponding normal tissues. * *p* < 0.05, ** *p* < 0.01, *** *p* < 0.001. Red boxes denote tumor tissues, while blue boxes represent normal tissues. The red highlights *SPP1* expression in tumor tissues, and the blue indicates *SPP1* expression in normal tissues. ACC: Adrenocortical carcinoma. BLCA: Bladder urothelial carcinoma. BRCA: Breast invasive carcinoma. CESC: Cervical squamous cell carcinoma and endocervical adenocarcinoma. CHOL: Cholangiocarcinoma. COAD: Colon adenocarcinoma. DLBC: Lymphoid neoplasm diffuse large B-cell lymphoma. ESCA: Esophageal carcinoma. GBM: Glioblastoma multiforme. HNSC: Head and neck squamous cell carcinoma. KICH: Kidney chromophobe. KIRC: Kidney renal clear cell carcinoma. KIRP: Kidney renal papillary cell carcinoma. LAML: Acute myeloid leukemia. LGG: Brain lower-grade glioma. LIHC: Liver hepatocellular carcinoma. LUAD: Lung adenocarcinoma. LUSC: Lung squamous cell carcinoma. MESO: Mesothelioma. OV: Ovarian serous cystadenocarcinoma. PAAD: Pancreatic adenocarcinoma. PCPG: Pheochromocytoma and paraganglioma. PRAD: Prostate adenocarcinoma. READ: Rectum adenocarcinoma. SARC: Sarcoma. SKCM: Skin cutaneous melanoma. STAD: Stomach adenocarcinoma. TGCT: Testicular germ cell tumors. THCA: Thyroid carcinoma. THYM: Thymoma. UCEC: Uterine corpus endometrial carcinoma. UCS: Uterine carcinosarcoma. UVM: Uveal melanoma.

We conducted a pan-cancer analysis of the *SPP1* expression utilizing TIMER2.0 (http://timer.cistrome.org/, obtained on 1 August 2024). The analysis indicated that the dysregulation of *SPP1* is prevalent across various tumor types, including breast cancer (BRCA). Specifically, *SPP1* was found to be upregulated in several cancers, including BLCA, BRCA, CESC, CHOL, COAD, ESCA, GBM, HNSC, KIRP, LIHC, LUAD, LUSC, PRAD, READ, STAD, THCA, and UCEC (Figure 2). Conversely, the *SPP1* expression was found to be downregulated in certain tumor types, such as KICH and KIRC (Figure 2). Additionally, the *SPP1* expression was significantly higher in metastatic SKCM compared to non-metastatic SKCM (Figure 2).

### 3.3. SPP1 mRNA Expression Is Positively Correlated with 21 Toll-like Receptor Signaling Pathway Genes Identified from the Three TNBC-Enriched Pathways

**Figure 3:**
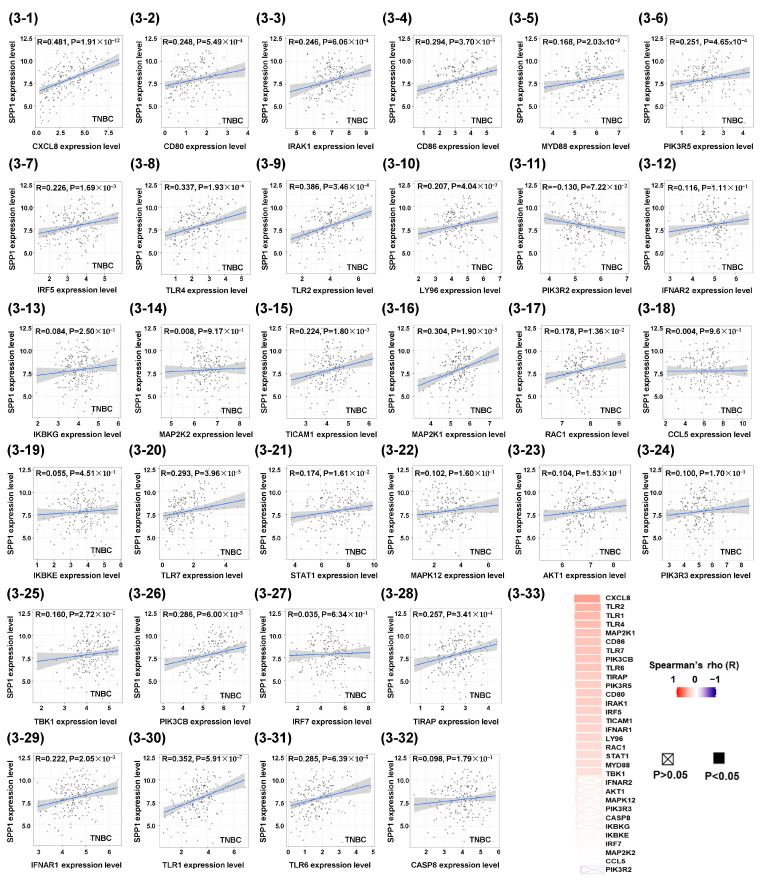
Spearman correlations between the expression of *SPP1* and the 32 Toll-like receptor signaling pathway genes in 191 TNBC patients.

Based on Figure 1, Spearman correlation analysis of the 32 genes in the Toll-like receptor signaling pathway gene set revealed that 21 were significantly associated with *SPP1* mRNA expression in a cohort of 191 TNBC patients in TIMER2.0 (Figure 3). Correlation plots for all 32 genes were generated, and their rho (R)-values and *p*-values were ranked and visualized using a heatmap (Figure 3).

**Figure 4:**
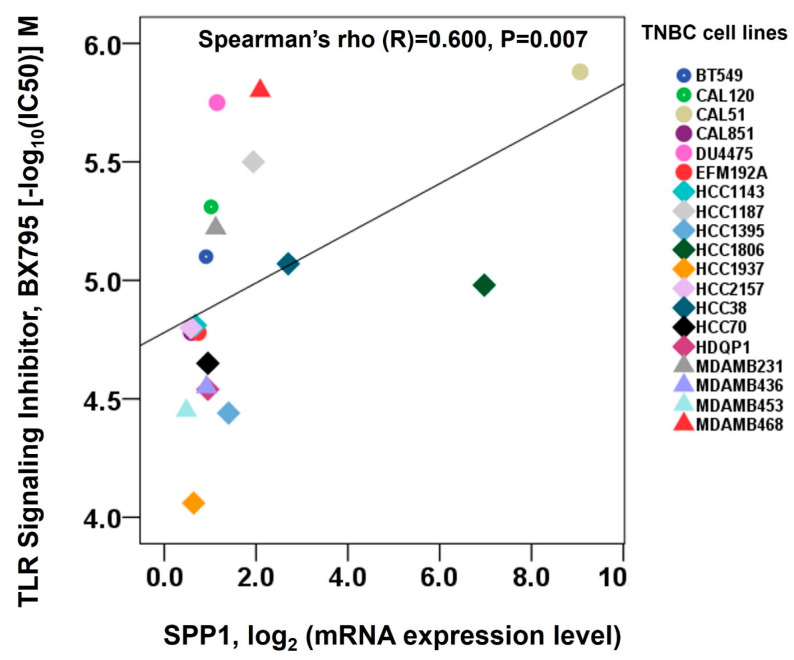
Spearman correlation between *SPP1* expression and BX795 sensitivity in TNBC cells.

To investigate the potential role of *SPP1* expression as a biomarker for Toll-like receptor (TLR) signaling pathway inhibition in triple-negative breast cancer (TNBC), a detailed analysis was conducted using Q-omics, a comprehensive platform integrating multi-omics data and drug-screening results (http://qomics.sookmyung.ac.kr/, data obtained on 10 November 2024). Among the compounds included in the Q-omics drug database, BX795, a known inhibitor of the TLR signaling pathway, was selected for evaluation. Through an integrative approach, this study assessed the relationship between the *SPP1* expression levels and the half-maximal inhibitory concentration (IC50) of BX795 across 19 different TNBC cell lines. The analysis revealed a positive correlation between the IC50 values of BX795 and *SPP1* expression, suggesting that higher *SPP1* expression levels are associated with reduced sensitivity to BX795 (Figure 4).

### 3.4. SPP1 mRNA in Survival Data

**Figure 5:**
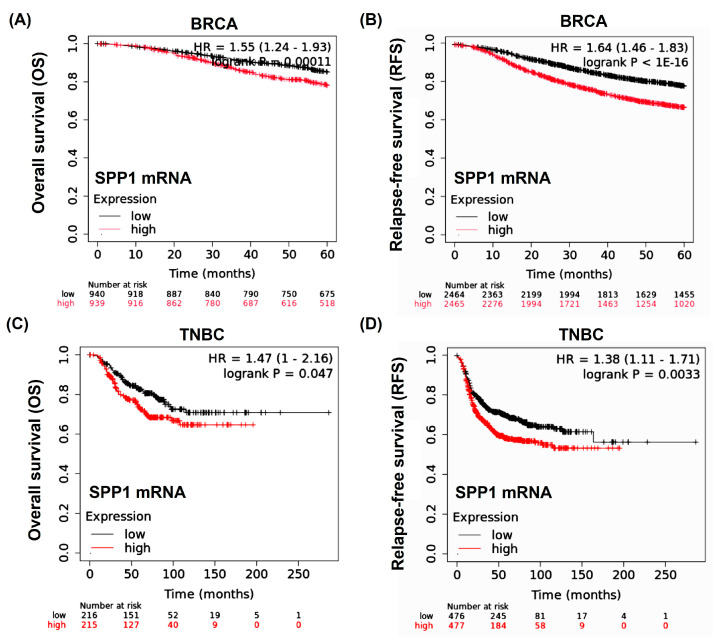
Higher *SPP1* mRNA expression had a poor prognosis in BRCA and TNBC. (**A**) Kaplan–Meier survival curve comparing the overall survival (OS) between the low and high *SPP1* mRNA expression cohorts in BRCA patients. (**B**) Kaplan–Meier survival curve comparing the relapse-free survival (RFS) between the low and high *SPP1* mRNA expression cohorts in BRCA patients. (**C**) Kaplan–Meier survival curve comparing the overall survival (OS) between the low and high *SPP1* mRNA expression cohorts in TNBC patients. (**D**) Kaplan–Meier survival curve comparing the relapse-free survival (RFS) between the low and high *SPP1* mRNA expression cohorts in TNBC patients.

In the Affymetrix GeneChip analysis (https://kmplot.com/analysis/, accessed on 1 August 2024), the overall survival (OS) rate at 20 years showed that high *SPP1* mRNA expression had a greater risk when compared to low *SPP1* BRCA (hazard ratio [HR] for death, 1.55; 95% confidence interval [CI], 1.24 to 1.93; log rank *p* = 0.00011) (Figure 5A). The rate of relapse-free survival (RFS) at 20 years was lower in the high *SPP1* expression BRCA as compared with the low *SPP1* expression BRCA (HR for recurrence or death, 1.64; 95% CI, 1.46 to 1.83; log rank *p* = 1.00×10^−16^) (Figure 5B). The OS rate at 20 years showed that high *SPP1* expression had a greater risk when compared to low *SPP1* TNBC (HR for death, 1.47; 95% CI, 1.00 to 2.16; log rank *p* = 0.047) (Figure 5C). The rate of RFS at 20 years was higher in low *SPP1* expression TNBC as compared with high *SPP1* expression TNBC (HR for recurrence or death, 1.38; 95% CI, 1.11 to 1.71; log rank *p* = 0.0033) (Figure 5D).

### 3.5. Expression of SPP1 Protein Is Downregulated in TNBC

**Figure 6:**
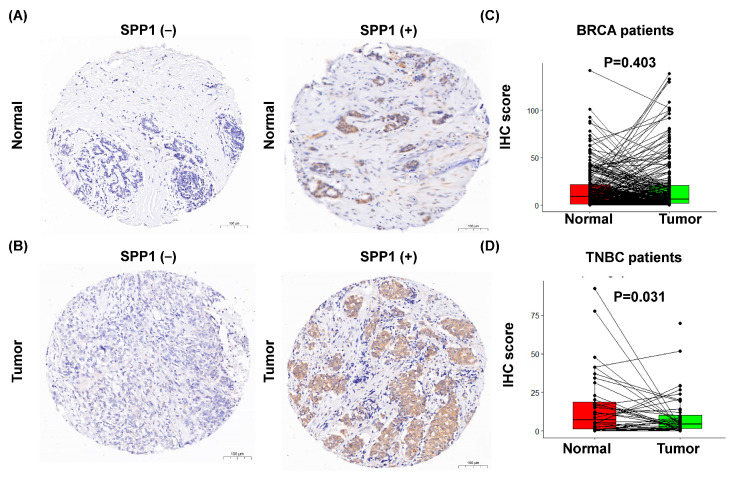
Immunohistochemical analysis of normal and tumor cell expression of SPP1 from representative samples (100×). Panel shows the number of samples evaluated by the specific SPP1 antibody and that they were positive for SPP1 expression (+) or negative (−). (**A**) The left panel shows representative results of low SPP1 immunostaining in normal breast tissue, while the right panel illustrates high SPP1 immunostaining. (**B**) The left panel displays representative results of low SPP1 immunostaining in breast tumor tissue, while the right panel shows high SPP1 immunostaining. (**C**) SPP1 is not significantly expressed in BRCA tissue compared to its matched normal breast tissue. (**D**) SPP1 is downregulated in TNBC compared to its corresponding normal breast tissues.

The association between SPP1 protein expression, BRCA, and TNBC survival prompted us to investigate the prognostic potential and clinical relevance of SPP1 protein expression in our breast cancer cohort study. We analyzed tissue samples from 282 independent patients, comparing the SPP1 protein expression levels between the breast tumors and their corresponding normal breast tissues using immunohistochemistry (IHC). The representative IHC staining for SPP1 is shown in Figure 6A,B. The SPP1 protein was mainly found at the cytoplasmic fraction of the tumor and normal tissues (Figure 6A,B). IHC showed no significant difference in SPP1 expression between BRCA tissues (mean ± SD: 18.41 ± 27.86) and normal breast tissues (mean ± SD: 16.64 ± 21.51) (*p* = 0.403, Figure 6C). However, SPP1 expression was significantly upregulated in TNBC tissues (mean ± SD: 15.76 ± 7.85) compared to normal breast tissues (mean ± SD: 7.85 ± 10.63) (*p* = 0.031, Figure 6D).

### 3.6. Stage, ER, PR, HER2, and BRCA Overall Survival

**Figure 7:**
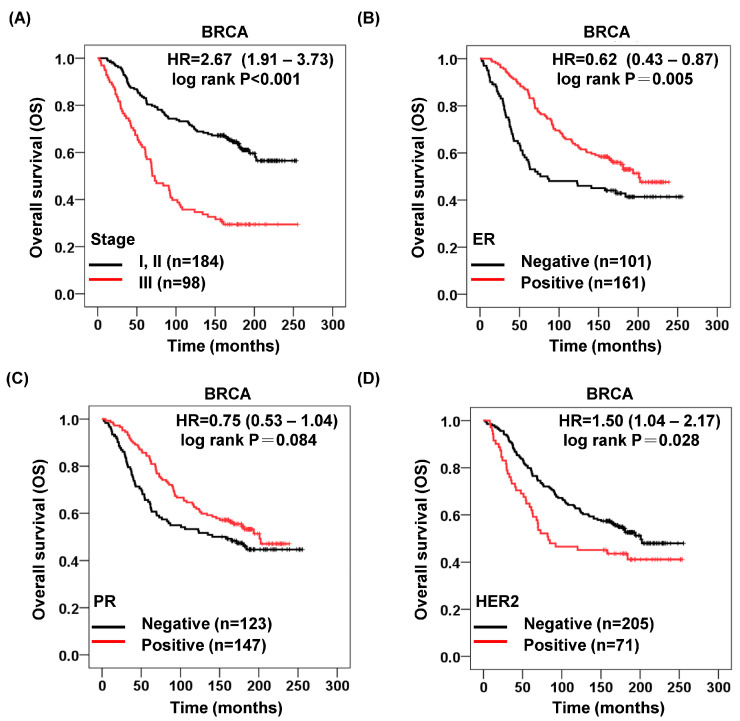
Late-stage, negative ER, and positive HER2 had a poor prognosis in BRCA. (**A**) Kaplan–Meier survival curve comparing the overall survival (OS) between the early- and late-stage cohorts in BRCA patients. (**B**) Kaplan–Meier survival curve comparing the overall survival (OS) between the ER-negative and ER-positive cohorts in BRCA patients. (**C**) Kaplan–Meier survival curve comparing the overall survival (OS) between the PR-negative and PR-positive cohorts in TNBC patients. (**D**) Kaplan–Meier survival curve comparing the overall survival (OS) between the HER2-negative and HER2-positive cohorts in TNBC patients.

As illustrated in Figure 7A, patients with stage III tumors in our cohort exhibited significantly poorer survival rates compared to those with stage I and II tumors (*p* < 0.001). Additionally, patients who tested positive for estrogen receptor (ER) expression demonstrated a notably improved survival rate in breast cancer compared to those with negative ER expression (*p* = 0.005, Figure 7B). Similarly, patients with positive progesterone receptor (PR) expression experienced a modest increase in survival rates compared to those with negative PR expression, although this difference did not reach statistical significance (*p* = 0.084, Figure 7C). Furthermore, higher levels of HER2 expression in tumor tissues were correlated with poorer survival outcomes in our cohort (*p* = 0.028, Figure 7D).

### 3.7. SPP1 Protein in Survival Data

**Figure 8:**
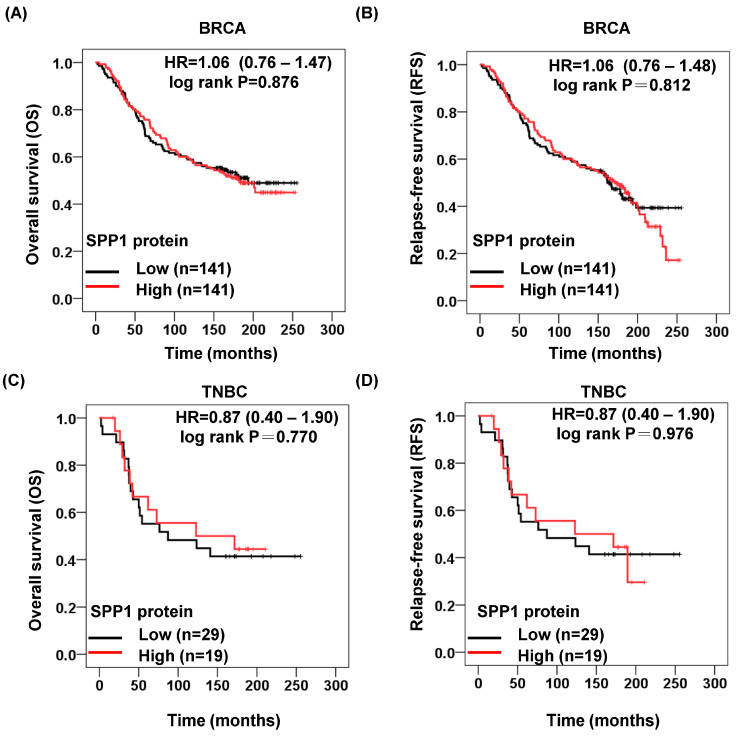
SPP1 protein expression had no prognostic significance in BRCA and TNBC. (**A**) Kaplan–Meier plot of the overall survival (OS) was applied to the low and high SPP1 protein cohorts in BRCA patients. (**B**) Kaplan–Meier plot of the relapse-free survival (RFS) was applied to the low and high SPP1 protein cohorts in BRCA patients. (**C**) Kaplan–Meier plot of the overall survival (OS) was applied to the low and high SPP1 protein cohorts in TNBC patients. (**D**) Kaplan–Meier plot of the relapse-free survival (RFS) was applied to the low and high SPP1 protein cohorts in TNBC patients.

The overall survival (OS) rate at 20 years showed that high SPP1 protein expression had no greater risk when compared to low SPP1 BRCA (HR for death, 1.06; 95% CI, 0.76 to 1.47; log rank *p* = 0.876) (Figure 8A). The rate of relapse-free survival (RFS) at 20 years was not higher in the high SPP1 BRCA as compared with in the low SPP1 expression BRCA (HR for recurrence or death, 1.06; 95% CI, 0.76 to 1.48; log rank *p* = 0.812) (Figure 8B). The OS rate at 20 years showed that high SPP1 expression had no greater risk when compared to low SPP1 TNBC (HR for death, 0.87; 95% CI, 0.40 to 1.90; log rank *p* = 0.812) (Figure 8C). The rate of RFS at 20 years was not higher in the high SPP1 expression TNBC as compared with in the high SPP1 expression TNBC (HR for recurrence or death, 0.98; 95% CI, 0.40 to 1.90; log rank *p* < 0.001) (Figure 8D).

### 3.8. Correlation Between M2 Macrophage and SPP1 Expression Level Is Statistically Significant in TNBC

**Figure 9:**
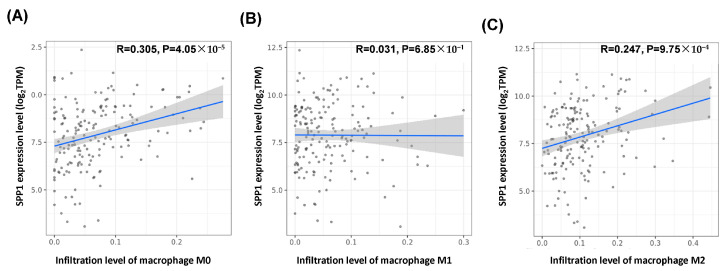
Associations between *SPP1* mRNA expression and the infiltration of different macrophage subtypes in TNBC: (**A**) M0 macrophages, (**B**) M1 macrophages, and (**C**) M2 macrophages.

To further investigate the relationship between the *SPP1* expression levels and macrophage infiltration, we used TIMER2.0. Notably, data from the CIBERSORT-ABS algorithm revealed a strong correlation between the *SPP1* and M0 macrophage infiltration levels in TNBC (Figure 9A). Additionally, *SPP1* showed a significant correlation with the M2 macrophage infiltration levels (Figure 9B), but no significant association with M1 macrophage infiltration in TNBC (Figure 9C).

## 4. Discussion

In this study, we focused on analyzing the correlation between *SPP1* expression and the prognosis of triple-negative breast cancer (TNBC). Initially, mRNA data from three TNBC tissue samples and their corresponding normal tissues were examined, revealing that genes involved in the Toll-like receptor signaling pathway were significantly upregulated in TNBC tissues compared to normal tissues. The *SPP1* expression in the TCGA TNBC dataset also demonstrated high levels (Figure 2), which were associated with a poor prognosis (Figure 5). This expression pattern was further confirmed at the SPP1 protein level in our own TNBC tissue cohort, showing no significance in relation to the TNBC survival rate. Additionally, *SPP1* expression was closely linked to tumor-associated macrophages (TAMs), specifically M2 macrophages, highlighting a significant association in TNBC (Figure 9B).

The Toll-like receptor (TLR) signaling pathway plays a crucial role in initiating immune responses and is expressed in both immune and tumor cells. It has been implicated in the progression of various malignancies, including breast cancer [17]. Targeting TLRs holds promise as a strategy to enhance breast cancer treatment, either as stand-alone therapies or in combination with existing treatments in breast cancer immunity and their potential in immunotherapy [18].

Our study revealed that elevated *SPP1* mRNA expression is associated with a poor prognosis; however, the immunostaining results indicated that SPP1 protein expression was localized exclusively in the cytoplasm and did not show a significant correlation with prognosis. The expression levels of *SPP1* mRNA have been identified as potential biomarkers for predicting recurrence in estrogen receptor-positive (ER+) breast cancer patients who have undergone tamoxifen treatment. This correlation suggests that monitoring *SPP1* mRNA expression may provide valuable insights into disease prognosis and aid in tailoring personalized treatment strategies for improved clinical outcomes. However, the expression of the SPP1 protein showed no correlation with either the risk of recurrence or the levels of *SPP1* mRNA [19].

Notably, immunostaining analyses in several prior studies highlighted that the nuclear isoform of SPP1 (OPN-c) is linked to poorer clinical outcomes, suggesting that the nuclear localization of this variant may play a critical role in influencing the prognosis [20]. A meta-analysis (Yu An et al., 2023) revealed the elevation of specific splice variants, namely SPP1-a, SPP1-b, and SPP1-c, in lung cancer and the elevation of SPP1-c in breast cancer as compared to healthy tissue [21]. The observation that alternative splice variants of *SPP1* may be produced selectively in cancer and that distinct types of cancer may express different combinations of spice variants has opened up the field for a more refined evaluation of the potential for utilizing SPP1 forms in cancer detection. In our study, immunohistochemistry (IHC) was performed using a primary mouse anti-human SPP1 monoclonal antibody (osteopontin/OPN/SPP1 antibody, AKm2A1; sc-21742, Santa Cruz Biotechnology, Inc.), which has previously been utilized in the immunohistochemical evaluation of osteopontin expression in triple-negative breast cancer, as described by Niedolistek et al. (2020) [22]. Variations in the changes and extent of the *SPP1* mRNA splicing across different regions of TNBC may also influence antibody engagement with antigenic epitopes differently.

*SPP1* expression in tumor-associated macrophages (TAMs) has drawn attention in various malignancies beyond lung cancer. In breast cancer, TAM-derived *SPP1* has been implicated in promoting cancer cell growth and progression. Single-cell RNA sequencing analysis has revealed that SPP1 is more highly expressed in monocyte-derived TAMs compared to resident macrophages/TAMs, further highlighting its role in tumor development [23]. Single-cell analysis in breast cancer has revealed that SPP1-positive tumor-associated macrophages (TAMs) display significantly increased expression of markers like apolipoprotein E (APOE), CD204, CD68, and CADM1. A high abundance of these SPP1-expressing TAMs, along with cancer-associated fibroblasts (CAFs), has been closely associated with resistance to immunotherapy. This suggests that the presence of these cells may contribute to a tumor microenvironment that hampers the effectiveness of immune-based treatments, highlighting their potential role in promoting therapy resistance [24]. Interestingly, a recent study by Liu et al. (2024) revealed a crucial mechanism underlying chemotherapy resistance in triple-negative breast cancer (TNBC) patients. The study identified a distinct population of macrophages that secrete secreted phosphoprotein 1 (SPP1), which interacts with CD44 receptors on the surface of TNBC cells. This interaction triggers a signaling cascade within the tumor cells, activating the intracellular PDE3B pathway via FYN-mediated integrin signaling [25]. Therefore, a thorough characterization of the phenotype and functional properties of the infiltrating macrophages in TNBC is vital for unraveling their contributions to tumor progression and mechanisms of therapy resistance.

In summary, this study builds upon previous reports highlighting the significant role of *SPP1* in cancer pathogenesis. The expression of *SPP1* mRNA may hold clinical relevance, as it is strongly associated with TNBC and patient survival outcomes. Our findings align with those of Gothlin Eremo et al., reinforcing the prognostic value of *SPP1* expression in TNBC. Additionally, we identify potential interacting proteins and enriched pathways that may mediate the tumor-promoting effects of *SPP1*. We also emphasize the relationship between *SPP1* and infiltrating macrophages in TNBC. However, our results show that the SPP1 protein does not reflect the outcome in TNBC patients. Thus, *SPP1* mRNA expression may be a better indicator of Toll-like receptor signaling upregulation, and thus, a predictive biomarker for the response to targeted therapies designed to address Toll-like receptor signaling.

## 5. Conclusions

Despite our study having limitations, such as a small sample size, incomplete clinical and pathological data, and the inherent heterogeneity of TNBC, it emphasizes the potential of *SPP1* mRNA as an important prognostic indicator and a promising molecular target for the treatment of TNBC.

## Figures and Tables

**Table 1:** NGS data were collected from three TNBC RNA samples and their corresponding normal RNA samples, with the NGS IDs linked to their clinical characteristics.

ID	Age	BMI	Menopausal Status	Grading	Stage	T	N	Survival
3	54	23.3	Peri-	III	IIa	T1a	N1	Alive
31	63	23	Post-	III	IIb	T2	N1	Death
39	77	23.5	Post-	II	IIIc	T2	N3	Death

NGS: next-generation sequencing. T: primary tumor. N: regional lymph node.

## Data Availability

The data presented in this study are available on request from the corresponding author.
